# Enhancing Executive Functions in Pediatric Epilepsy: Feasibility and Efficacy of a Computerized Cognitive Training Program

**DOI:** 10.3390/children11040484

**Published:** 2024-04-18

**Authors:** José Luis Tapia, Luis Miguel Aras, Jon Andoni Duñabeitia

**Affiliations:** 1 Centro de Investigación Nebrija en Cognición (CINC), Universidad Nebrija, 28043 Madrid, Spain; jtapia@nebrija.es; 2 Servicio Navarro de Salud, 31008 Pamplona, Spain; luismi.aras@apoyodravet.eu; 3 Department of Languages and Culture, UiT The Arctic University of Norway, 9019 Tromsø, Norway

**Keywords:** pediatric epilepsy, cognitive training, executive functions, inhibitory control, computerized intervention, cognitive rehabilitation

## Abstract

Epilepsy, a prevalent neurological disorder characterized by recurrent seizures, significantly impacts individuals’ neurobiological, cognitive, and social lives. This report presents a feasibility study investigating the effects of a computerized cognitive training program on enhancing executive functions, particularly inhibitory control, in children and adolescents with epilepsy. Employing a pre-test–intervention–post-test design, the study involved 26 participants with diverse epileptic syndromes, focusing on those without severe intellectual disabilities. The intervention, based on the CogniFit Inc. platform, consisted of personalized tasks aiming to improve participants’ inhibitory skills over 16 weeks, with an average of 40 sessions completed per participant. Results indicated significant improvements in reaction times and error rates in an anti-saccade task, demonstrating enhanced inhibitory control and general performance post-intervention. These findings suggest that targeted cognitive training is a feasible approach to bolster executive functions in young individuals with epilepsy, potentially improving their academic performance, employability, and social interactions. The study underscores the importance of early cognitive interventions in epilepsy management, highlighting the potential for computerized programs to aid in mitigating cognitive deficits associated with the condition.

## 1. Introduction

Epilepsy stands as one of the most prevalent chronic neurological conditions worldwide, primarily characterized by the occurrence of recurrent seizures stemming from abnormal electrical discharges in the brain. Notably, in 2010, the World Health Organization classified epilepsy as the second most significant neurological disorder [1]. The prevalence of epilepsy is influenced by demographic factors such as age, gender, ethnicity, and geographic location [2,3], revealing the condition’s complexity and the diverse populations it affects. Advances in perinatal care and health services have led to a noticeable decrease in early-life epilepsy. However, adolescence and early adulthood remain the periods with the highest prevalence [4].

Pediatric epilepsy, distinct in its etiology and impact, significantly disrupts neurodevelopment and affects the entire spectrum of physical, cognitive, and emotional health [5]. Research has shown a notable decrease in higher cognitive functions in patients with pediatric epilepsy compared to individuals without this condition. Deficiencies in various cognitive skills, particularly those involving executive functions, have been identified across multiple studies [6,7,8].

### 1.1. The Importance of Executive Functions

Executive functions represent an essential set of higher cognitive skills that orchestrate the control and regulation of human behavior [9]. They include processes such as planning, organization, decision-making, problem-solving, working memory, cognitive flexibility, inhibition, and attention. These abilities are vital for adapting and efficiently managing daily demands, enabling individuals to plan future actions, organize tasks, make informed decisions, solve problems deliberately, set goals, and develop strategies to achieve them. The development of executive functions is intrinsically linked to the maturation of brain structures, including the prefrontal cortex and associated regions of the parietal lobe, temporal lobe, and limbic system. Childhood and adolescence mark a critical period in this evolutionary process, characterized by rapid advancements in these cognitive skills, which later stabilize during young adulthood [10], and then start a progressive age-associated decline [11].

Within the spectrum of executive functions, inhibition emerges as a key competency, critical for proper daily functioning and cognitive control. Inhibition is defined as the ability to suppress impulsive responses, enabling the generation of behaviors mediated by conscious processes of attention and reasoning. Cognitive inhibition has been identified as a significant predictor of academic performance [12], can prevent engagement in risky behaviors [13,14], and contributes to the achievement of long-term goals [15]. Given its significant relevance for individual independence and autonomous functioning, early neuropsychological assessment seems vital for detecting potential cognitive impairments and enabling targeted rehabilitation interventions.

To date, a variety of assessment tools have been utilized to evaluate cognitive impairment associated with epilepsy, including tests focused on attention, language, memory, processing speed, and executive functions, among other aspects. As for the specific assessment of inhibition, the anti-saccade is frequently employed in the literature of psychology and cognitive neuroscience to investigate deficiencies in inhibitory control, assessing the ability to suppress an automatic or prepotent ocular response in favor of a controlled and deliberate voluntary action [16]. A study that included children with epilepsy utilized functional magnetic resonance imaging (fMRI) to analyze performance on the anti-saccade task. Despite observing comparable neural pathway activation between children with epilepsy and healthy controls, the former group committed a higher number of errors in the anti-saccade responses [17].

### 1.2. Cognitive Intervention for Epilepsy

Currently, cognitive stimulation, and in more detail computerized cognitive training, is emerging as a valuable therapeutic tool to address cognitive performance. This method aims to recover, compensate, or enhance cognitive functions through training or the repetitive practice of specific strategies [18]. Its efficacy in enhancing cognitive mechanisms in individuals with epilepsy is particularly promising, considering the proven utility of cognitive stimulation programs in rehabilitating executive functions in disorders such as Attention Deficit Hyperactivity Disorder (ADHD) [19,20], Autism Spectrum Disorder (ASD) [21], insomnia [22], cancer-related cognitive impairment (CRCI) [23], post-acute sequelae of COVID-19 (PASC) [24], and schizophrenia [25], among others.

A recent study comparing computer-based to task-oriented cognitive rehabilitation programs in children with epilepsy showed significant benefits in both approaches, surpassing the control group that received no intervention [26]. These programs included 12 sessions of approximately 45 min each, highlighting the positive impact of well-designed interventions on the cognitive development of children with epilepsy. Additionally, the growing popularity of digital and gamified interventions, noted for their accessibility, personalization, and high adherence [27], underscores the evolution of therapeutic strategies in the treatment of epilepsy. In this context, Engelberts et al. [28] documented improvements in self-reported cognitive performance and quality of life in outpatients with focal seizures and attention deficits, although no significant differences were found in tests of inhibitory capacity. Complementarily, Helmstaedter et al. [29] implemented a computerized training program designed to improve attention, memory, and executive functions in patients who had undergone temporal lobe surgery. This program, which included a total of six tasks adjustable in difficulty based on user performance, demonstrated a significant positive impact on verbal learning and memory, despite being a short-term intervention of 30 days. Similarly, Koorenhof et al. [30] applied a computerized training focused on memory, mental flexibility, cognitive control, and processing speed in epileptic adults before and after left temporal lobectomy. The training regime, consisting of 40 sessions of 15 min each, resulted in a general improvement in the performance and mood of the participants, although no significant differences were found in the domain of memory. Despite the relevance of these findings, the literature on cognitive training in the context of pediatric epilepsy remains insufficient [31].

### 1.3. Our Proposal

Acknowledging the crucial importance of executive functions and inhibitory capacity in daily-life performance, this feasibility study aims to test the effectiveness of a computerized cognitive training intervention in young individuals affected by epilepsy. This program will feature short-duration sessions, designed to maximize adherence and effectiveness by increasing the total number of sessions. By focusing on the young population, this study seeks not only to evaluate the efficacy of the cognitive intervention in this specific group but also to provide valuable insights on the best ways to support their cognitive development and enhance their quality of life in the long term. Changes as a function of the intervention will be measured with a children-friendly behavioral version of the anti-saccade task.

## 2. Methods

### 2.1. Participants

This study involved a total of 26 children and adolescents diagnosed with pediatric epilepsy, encompassing a diverse range of epileptic syndromes and seizure foci. The participants’ ages ranged from 7 to 17 years, with a mean age of 12.11 years (SD = 2.96). Of these, 12 were female, reflecting a balanced gender distribution within the sample. Except for one participant residing in Colombia, all participants were from various provinces of Spain, ensuring a primarily Spanish-speaking cohort. All participants had Spanish as their native language. This homogeneity in language was crucial for standardized administration and understanding of the intervention and assessment tasks. A power analysis was conducted using G*Power (v.3.1.9.7) to determine the required sample size, assuming a medium effect size of f^2^ = 0.35, an alpha error probability of 0.05, and a power (1—β error probability) of 0.80. The analysis indicated that a total sample size of 25 participants would be necessary to achieve a power of approximately 0.81 for detecting the presumed effect.

Individuals were considered for the study if they met the following criteria: (1) they were Spanish-speaking minors aged between 6 and 18 years, (2) they had been diagnosed with epilepsy for at least 12 months before showing interest in the study, and (3) they did not exhibit cognitive symptoms that would prevent them from participating in the tasks and training. A trained neuroscientist on the research team reviewed all applications and selected candidates according to these criteria, disqualifying those who did not qualify. Detailed information regarding cognitive skills was collected from medical reports and disability assessments provided by the families. In instances with insufficient data or unclear cases, intellectual disability classifications were made based on communication skills and overall functioning during a consensus meeting led by a neuropsychologist and the second author. Three exclusion criteria were predefined for the accepted participants: (1) completion of both the initial and final cognitive assessment tasks, (2) attendance of at least 10 training sessions, and (3) achieving a minimum average accuracy of 50% in the cognitive assessment tasks. Among the 34 individuals interested, 1 was not included due to age not falling within the required range. Of the 33 participants, 7 were removed from the final analysis because 3 failed to attend the minimum number of sessions required, and 4 did not complete the final assessment. The data of the 26 participants who passed the inclusion criteria and did not meet any exclusion criteria were analyzed. Detailed characteristics of the sample are provided in Table 1.

All participants were screened to exclude any moderate or severe intellectual or cognitive disability, allowing for a focused examination of the intervention’s effect on children and adolescents whose cognitive development was within the expected range for their age, notwithstanding their epilepsy diagnosis. The types of epilepsy represented in the study were notably varied, including but not limited to two cases of Dravet Syndrome and two cases of Rolandic epilepsy, highlighting the intervention’s applicability across different epileptic conditions.

Ethical considerations were rigorously adhered to, with informed consent obtained from all participants’ primary caregivers and legal tutors. This consent process was conducted with full disclosure of the study’s protocol, ensuring that families were well informed about the nature, potential benefits, and risks of participation. The study was executed in accordance with current regulations and the ethical principles outlined in the Declaration of Helsinki, receiving approval from the University of Nebrija Ethics Committee under the code number UNNE-2023-0006.

### 2.2. Materials and Procedure

Two main types of materials were used: the assessment task used in the pre- and post-test, and the intervention protocol used during the 16 weeks between assessments. Thus, the study followed a pre-test–intervention–post-test design without a control group.

**Figure 1:**
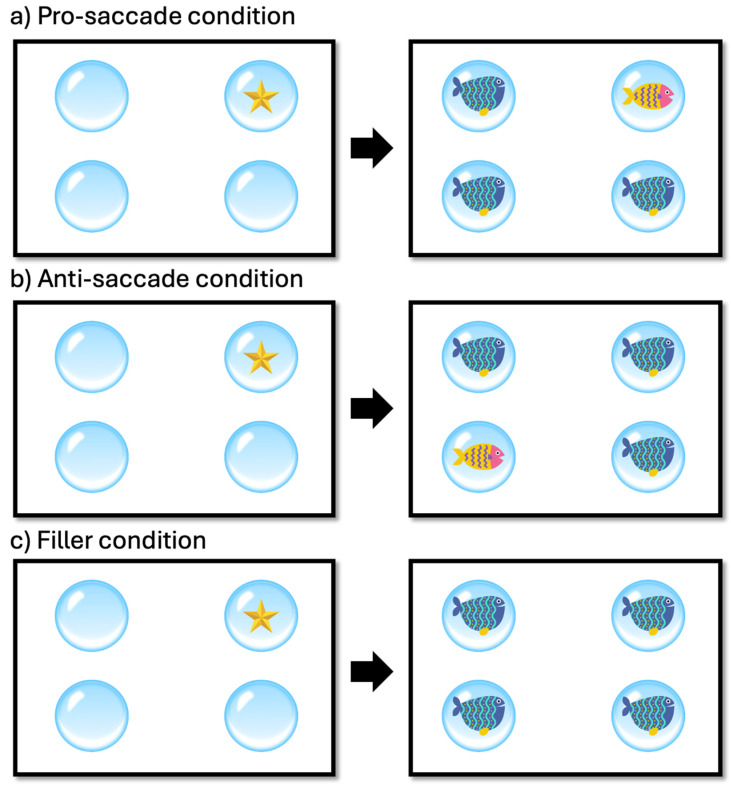
Examples of the three experimental conditions used in the anti-saccade task.

For the assessment of inhibitory control, an adapted version of the anti-saccade test from the DIGICOG executive function battery (Impulso Cognitivo SL, Ermua, Spain) was used. This child-friendly version was designed to be engaging and accessible. The test included visual stimuli presented in four bubbles arranged in each of the quadrants of a computer screen, featuring two types of fish characters serving as target and filler stimuli. The task also incorporated a pro-saccade and anti-saccade condition to assess the participants’ ability to inhibit reflexive glances towards a cue and instead focus on the target stimulus. To this end, in all trials, a star would appear immediately before the target or filler fish (see Figure 1). In the case of a target fish appearing on the screen, the star could have appeared either in the same exact bubble (pro-saccade condition), or in the opposite bubble (anti-saccade condition). Participants were instructed to disregard this star and not to respond to it, and they were told to press the appropriate button based on whether they saw the target fish (a yellow fish with purple stripes and a pink face) in any of the bubbles, or they only saw filler fish in all the bubbles (a blue fish with light blue stripes and yellow dots; see Figure 1 for details).

**Figure 2:**
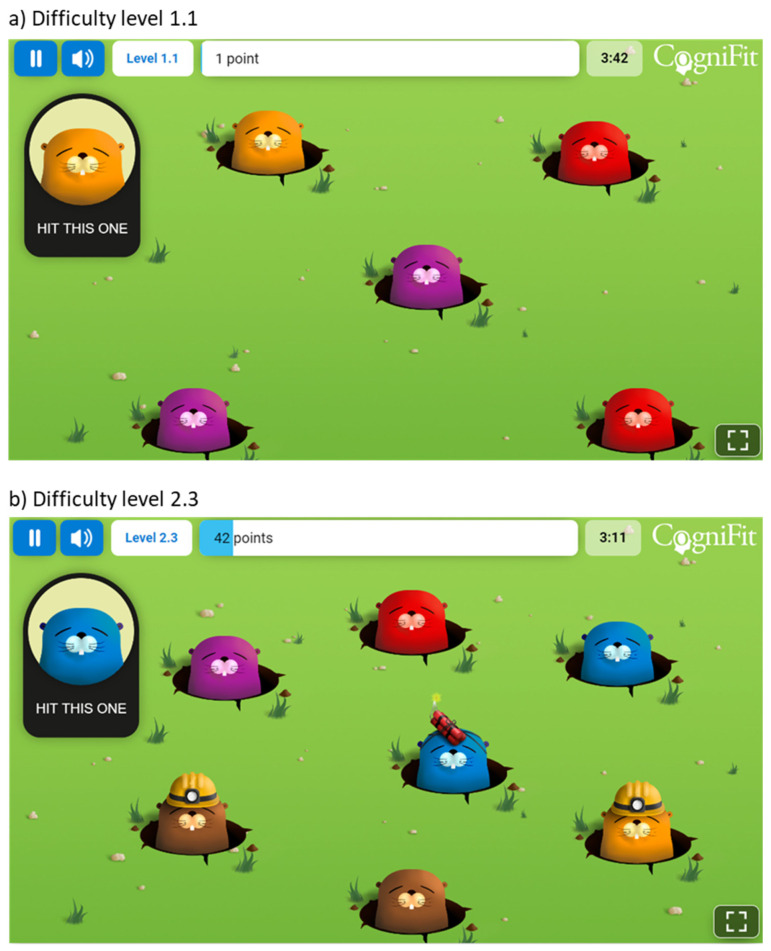
Example of a training activity used in the cognitive intervention (taken from CogniFit^®^ app, version 4.4).

The task structure included 8 practice trials to familiarize participants with the task requirements and stimuli, and 64 experimental trials to assess inhibitory control under different conditions (i.e., 32 filler trials, 16 trials of pro-saccade condition, 16 trials of anti-saccade condition). The timing of the task was carefully designed to measure rapid visual processing and response inhibition, with a sequence of stimuli presentation that required participants to respond quickly and accurately to the target fish while ignoring the distracting stars. Each trial began with the presentation of a fixation cross at the center of the screen, lasting for 500 milliseconds. This served to orient the participants’ attention to the center of the screen, preparing them for the stimulus onset. Following the fixation cross, the screen displayed the four bubbles for 500 ms without any other visual information. Next, the star would be presented for 500 ms in one of the four bubbles. Immediately following the star, the target or filler fish appeared. The fish remained on the screen until the participant responded by pressing the appropriate button or for a maximum duration of 4000 ms. Upon completion of the initial assessment with the anti-saccade test, participants began the training period, which lasted for 16 weeks. Each training session lasted for about 12–15 min, and participants were instructed to complete a minimum of 3 training sessions per week. The intervention utilized a computerized cognitive training module provided by CogniFit Inc. (San Francisco, CA, USA). This personalized training aimed at enhancing participants’ inhibitory skills, a core component of executive functions. The training encompassed a variety of games and tasks, starting at the lowest difficulty level. The Individualized Training System™ (ITS; CogniFit Inc., San Francisco, CA, USA) algorithm dynamically adjusted the difficulty based on each participant’s performance, ensuring a tailored training experience. One such activity, titled “Reaction Field”, is depicted in Figure 2, showcasing a meadow where various moles appear, and the participant is instructed to select the mole that matches the example shown in the top-left corner of the screen. Moles briefly emerge and either disappear after a short time or when the participant correctly selects the target. The activity begins at the lowest difficulty, escalating in complexity through 10 levels as the participant achieves the set objectives. The panel (a) illustrates the activity at a starting difficulty of 1.1, with a limited number of moles presented. Panel (b) progresses to difficulty level 2.3, increasing the total number of moles and introducing additional challenges: some moles are wearing helmets, requiring a double selection, and others are equipped with dynamite, which removes the mole if selected. Participants engaged in the training sessions at home, under the supervision of their primary caregivers. A trained psychologist provided continuous support throughout the assessment and training process, ensuring adherence, and addressing any questions or concerns.

After the 16th week, participants underwent a post-test using the same anti-saccade task to evaluate changes in their inhibitory control abilities. This design allowed for the assessment of the training’s effectiveness in enhancing core components of executive functions among the participants.

Parents and legal guardians were notified that they could reach out to the research team if they observed any side effects following the training sessions. If any unexpected effects, whether harmful or adverse, were noticed following the computerized cognitive training, participants and their families were advised to promptly contact the researchers. If any safety concerns were reported, a medical professional on the research team would swiftly conduct a video interview to assess the severity of the side effect or adverse event. This assessment would utilize criteria and elements from classic patient-reported adverse event questionnaires, e.g., [32]. It is important to note that no participants or their guardians reported any unintended effects from the cognitive training.

## 3. Results

The commitment to the intervention was significant, with participants completing an average of 40.23 training sessions (SD = 17.82), translating to a mean total training time of 356 min (SD = 221). This level of engagement indicates a substantial investment of time and effort by the participants and their families in the cognitive training process.

The analysis of the reaction time and accuracy data (measured in terms of percentages of errors) associated with the anti-saccade assessment tasks completed at pre- and post-test followed a 2 (Experimental Condition: pro-saccade, anti-saccade) × 2 (Time of Measurement: pre-test, post-test) design. Differences between the levels of Time of Measurement in the responses to the filler items were analyzed separately, given that they required a different response. All the analysis was carried out using jamovi [33], an open-source statistical software that integrates with the R programming language [34], employing the General Analyses for Linear Models (GAMLj) module [35] for our computations.

### 3.1. Reaction Time Analysis

First, all responses associated with erroneous decisions were discarded. Next, all reaction times deviating in more than two standard deviations from the mean of each participant in each condition were also discarded (4.76% of the data). The remaining datapoints were analyzed using a linear mixed model approach, with the two main factors and their interaction as fixed factors, and a random intercept for the participants. Note that more complex fixed and random structures were also tested, including factors related to the number of intervention sessions per participant and all possible interactions and random slopes, but the one finally used was the model providing lower BIC and AIC. The results of the omnibus test for the fixed factors using the Satterthwaite method for the calculation of degrees of freedom showed a significant effect of Time of Measurement [F(1, 1365) = 13.12, *p* < 0.001], demonstrating that participants responded to the items faster in the post-test than in the pre-test (a 126 ms difference; 1735 vs. 1861 ms, respectively; see Table 1). The analysis also showed significant differences between the levels of Experimental Condition [F(1, 1368) = 6.52, *p* = 0.011], indicating that participants took longer in responding to trials in the anti-saccade condition than in the pro-saccade condition (a 93 ms difference; 1847 vs. 1754 ms, respectively; see Table 1). The interaction between the two factors was not significant [F < 1.3 and *p* > 0.25]. The analysis of the latency data associated with the filler trials showed a significant effect of Time of Measurement [F(1, 1370) = 10.8, *p* = 0.001], with a 143 ms difference between latencies at pre-test and post-test (1899 vs. 1756 ms, respectively). In total, 19 out of the 26 participants improved their overall performance as measured by reaction times across experimental conditions after training, representing 73% of the sample.

### 3.2. Error Rate Analysis

Given the binary nature of the responses, the analysis was carried out using a generalized mixed model. For coherence purposes, the same structure used in the reaction time analysis was used. The fixed effects omnibus test showed a significant effect of Experimental Condition [χ^2^(1) = 24.46, *p* < 0.001], showing that items in the anti-saccade condition yielded higher error rates than those in the pro-saccade condition (a 7.4% difference; 16.8% vs. 9.4%, respectively; see Table 2). The main effect of Time of Measurement and the interaction between the two factors were not significant [χ^2^ < 1.5 and ps > 0.22]. The analysis of the error rates in the non-target condition did not show any significant difference between pre-test and post-test [χ^2^(1) = 0.76, *p* = 0.38].

## 4. Discussion

This study aimed to evaluate the effects of a computerized 16-week cognitive training program on executive functioning, and in more detail on cognitive control and inhibitory capacities, in children and adolescents with pediatric epilepsy. By using a child-friendly modified version of the anti-saccade task as a pre-test and post-test measure, the efficacy of the cognitive training was assessed.

The results of the study revealed three pivotal findings: First, the use of anti-saccade trials effectively discriminated cognitive control and inhibitory capabilities in children and adolescents with pediatric epilepsy, as evidenced by longer reaction times and higher error rates compared to pro-saccade trials. This indicates the task’s validity in measuring intended cognitive functions in this population. Second, participants exhibited significant adherence to the computerized cognitive training, suggesting its suitability and appeal for the pediatric population. Third and most crucially, the training led to substantial improvements in task performance, marked by reduced reaction times, thereby demonstrating enhanced cognitive control following the intervention. This decrease in reaction times after the intervention highlights the effectiveness of this computerized training in augmenting cognitive control, aligning with the theoretical models that suggest that cognitive control and inhibitory processes are adaptable and can be enhanced through focused intervention [36,37].

Our results add to the growing body of evidence demonstrating the efficacy of cognitive training interventions in juvenile populations affected by neurological illnesses when viewed in the light of the body of current literature. Research like that conducted in the context of ADHD [38] has shown that children executive function deficiencies can significantly improve their cognitive performance by completing tasks that are intended to target specific components of the executive function network. Our findings expand on this body of work by showing comparable advantages in children with epilepsy, a population that is in desperate need of cognitive therapies but has received little scientific attention.

Furthermore, our study’s observation of the distinct effects of pro- and anti-saccade trials provides important new information regarding the nature of the cognitive control difficulties that epileptic children specifically face. The longer reaction times and higher error rates in anti-saccade trials underscore the heightened difficulty in overriding prepotent responses, a core aspect of inhibitory control. This result is in line with proposals emphasizing the importance of inhibitory control in supporting more general cognitive and social abilities, indicating that gains in this fundamental ability may have significant effects on children with epilepsy [9,39,40].

The current study does have several drawbacks, though. It is difficult to attribute changes to the cognitive training intervention alone because there is no control group and the participants’ types of pediatric epilepsy varied widely. Neuroimaging studies have demonstrated atypical development in the frontal lobe that is seemingly independent of the seizure-affected areas of the brain area [41,42], implying that cognitive functions reliant on frontal neural circuits may be inherently altered, regardless of seizure type. Furthermore, while antiepileptic medication is often linked to cognitive decline [43,44], there is mounting evidence that cognitive impairments can manifest prior to treatment [45,46,47,48]. Indeed, some pharmacological treatments have been observed to enhance executive functions [49]. In addition to the vast variability in the etiologies of epilepsy, there is also a high rate of change in pharmacological regimens. It is commonplace to alter both the medication and dosage frequently until the symptoms are effectively managed [50]. We encountered a significant challenge regarding the collection of detailed pharmacological data for the pediatric participants diagnosed with epilepsy. Notably, a majority of parents or legal guardians (14 out of 26) chose not to disclose specific information about the antiepileptic drugs and whether their children were undergoing polytherapy or monotherapy. This limitation, while noteworthy, did not detract from our research objective, which was to assess the feasibility of a computerized cognitive intervention for a broad spectrum of pediatric epilepsy cases. We approached our study with the understanding that our participants represented a diverse group in terms of epilepsy types and comorbid conditions. These drawbacks emphasize the need for additional studies using more reliable experimental designs, such as stratified epilepsy types and randomized controlled trials, to determine the effectiveness and generalizability of cognitive training interventions more conclusively in this population. The impact of epilepsy on cognitive function can vary widely, depending on factors such as the type of epilepsy, the age at onset of the disease, the location and lateralization of the seizures, the pharmacological treatment, as well as the individual characteristics of the patient. Therefore, a comprehensive neuropsychological and developmental approach is crucial for understanding and managing the cognitive alteration associated with epilepsy.

## 5. Conclusions

This study offers encouraging proof that computerized cognitive training can improve executive functioning in children and adolescents with pediatric epilepsy. The significant improvements observed post-intervention demonstrate the possibility of these training regimens to lessen the cognitive impairments frequently linked to epilepsy. Notwithstanding the drawbacks, these results represent a significant advancement in the effort to create efficient, empirically supported therapies for cognitive rehabilitation in children with epilepsy. Building on these preliminary findings, future research should strive to resolve methodological constraints and broaden the scope to investigate long-term consequences and practical usefulness.

## Figures and Tables

**Table 1:** Summary of demographic and clinical characteristics of the study participants.

ID	Gender	Age	Condition	Associated Condition(s)
1	Male	9	Refractory focal epilepsy	ADHD Combined
2	Male	11	Epilepsy	ADHD
3	Female	8	Epilepsy	-
4	Female	16	Dravet Syndrome	-
5	Female	9	Epilepsy due to PCDH19 gene mutation	-
6	Male	10	Focal epilepsy	Dyslexia and dysgraphia
7	Male	13	Refractory epilepsy	-
8	Male	16	Dravet Syndrome	-
9	Male	13	Dravet Syndrome	-
10	Male	12	Idiopathic epilepsy	Non-verbal learning disorder
11	Female	14	Epilepsy	ASD features, Genetic Syndrome
12	Female	16	Refractory epilepsy	ASD features
13	Male	7	Epilepsy	CSWS, Polymicrogyria, ADHD
14	Male	15	Refractory epilepsy	Double Hit, Craniotomy
15	Male	14	Rolandic epilepsy	-
16	Female	11	Generalized epilepsy	-
17	Male	13	Epilepsy	Attention deficit
18	Male	15	Epilepsy	-
19	Female	9	Focal epilepsy	-
20	Male	9	Refractory focal epilepsy	Cerebral palsy without cognitive impairment and with autonomous gait, ADHD of inattentive type, visual hallucinations (occipital focus)
21	Male	10	Atypical Rolandic epilepsy	-
22	Female	17	Epilepsy	-
23	Female	13	Epilepsy	Ring chromosome 20 mutation, OCD, attention deficit
24	Female	16	Refractory epilepsy	Genetic alteration of the SCN2A gene, ASD
25	Female	9	Refractory epilepsy	ADHD, dyslexia, dyscalculia
26	Female	10	Refractory epilepsy	Congenital CMV infection, bilateral hearing loss with cochlear implants, right hemiparesis

Note. ADHD = Attention Deficit/Hyperactivity Disorder; ASD = Autism Spectrum Disorder; CSWS = Continuous Spikes and Waves during Slow sleep; OCD = Obsessive Compulsive Disorder; CMV = Cytomegalovirus.

**Table 2:** Mean reaction times (in ms) and percentages of errors (in parentheses) in all experimental conditions across times of measurement.

				95% Confidence Interval	
Experimental Condition	Time of Measurement	Mean	SD	Lower	Upper
Anti-saccade	Post-test	1763 (17.5%)	664 (38.1)	1691 (13.9)	1835 (21.2)
Pro-saccade	Post-test	1708 (10.3%)	583 (30.5)	1648 (7.4)	1769 (13.3)
Filler	Post-test	1756 (12.1%)	633 (32.7)	1709 (9.9)	1803 (14.4)
Anti-saccade	Pre-test	1929 (16.1%)	782 (36.8)	1845 (12.6)	2013 (19.7)
Pro-saccade	Pre-test	1798 (8.4%)	703 (27.8)	1726 (5.7)	1871 (11.1)
Filler	Pre-test	1899 (13.3%)	746 (34.0)	1843 (11.0)	1954 (15.7)

## Data Availability

The data that support the findings of this study are available from the authors upon request. The data are not publicly available due to restrictions imposed to preserve individuals’ privacy.
